# A Complete Digital Workflow for Planning, Simulation, and Evaluation in Orthognathic Surgery

**DOI:** 10.3390/jcm10174000

**Published:** 2021-09-03

**Authors:** Sang-Jeong Lee, Ji-Yong Yoo, Sang-Yoon Woo, Hoon Joo Yang, Jo-eun Kim, Kyung-Hoe Huh, Sam-Sun Lee, Min-Suk Heo, Soon Jung Hwang, Won-Jin Yi

**Affiliations:** 1 Dental Research Institute, Seoul National University, Seoul 03080, Korea; sjlee89@snu.ac.kr; 2 Department of Biomedical Radiation Sciences, Graduate School of Convergence Science and Technology, Seoul National University, Seoul 08826, Korea; gd_yoo@snu.ac.kr (J.-Y.Y.); woodli14@snu.ac.kr (S.-Y.W.); 3 Department of Oral and Maxillofacial Surgery and Dental Research Institute, School of Dentistry, Seoul National University, Seoul 03080, Korea; 4 Department of Oral and Maxillofacial Radiology, Seoul National University Dental Hospital, Seoul 03080, Korea; noel1st@snu.ac.kr; 5 Department of Oral and Maxillofacial Radiology and Dental Research Institute, School of Dentistry, Seoul National University, Seoul 03080, Korea; future3@snu.ac.kr (K.-H.H.); raylee@snu.ac.kr (S.-S.L.); hmslsh@snu.ac.kr (M.-S.H.); 6 Hwang Soon Jung’s Dental Clinic for Oral and Maxillofacial Surgery, Seoul 06626, Korea; sjhwang@snu.ac.kr

**Keywords:** digital workflow, orthognathic surgery, cloud-based collaboration, digital natural head position reproduction, 3D landmark-based evaluation

## Abstract

The purpose of this study was to develop a complete digital workflow for planning, simulation, and evaluation for orthognathic surgery based on 3D digital natural head position reproduction, a cloud-based collaboration platform, and 3D landmark-based evaluation. We included 24 patients who underwent bimaxillary orthognathic surgery. Surgeons and engineers could share the massive image data immediately and conveniently and collaborate closely in surgical planning and simulation using a cloud-based platform. The digital surgical splint could be optimized for a specific patient before or after the physical fabrication of 3D printing splints through close collaboration. The surgical accuracy was evaluated comprehensively via the translational (linear) and rotational (angular) discrepancies between identical 3D landmarks on the simulation and postoperative computed tomography (CT) models. The means of the absolute linear discrepancy at eight tooth landmarks were 0.61 ± 0.55, 0.86 ± 0.68, and 1.00 ± 0.79 mm in left–right, advance–setback, and impaction–elongation directions, respectively, and 1.67 mm in the root mean square direction. The linear discrepancy in the left–right direction was significantly different from the other two directions as shown by analysis of variance (ANOVA, *p* < 0.05). The means of the absolute angular discrepancies were 1.43 ± 1.06°, 0.50 ± 0.31°, and 0.58 ± 0.41° in the pitch, roll, and yaw orientations, respectively. The angular discrepancy in the pitch orientation was significantly different from the other two orientations (ANOVA, *p* < 0.05). The complete digital workflow that we developed for orthognathic patients provides efficient and streamlined procedures for orthognathic surgery and shows high surgical accuracy with efficient image data sharing and close collaboration.

## 1. Introduction

Orthognathic surgery is used in the correction of facial asymmetry, dentofacial deformity, and skeletal malocclusion, as well as adjunctive procedures, to improve masticatory function and esthetics. In conventional planning for orthognathic surgery, surgeons use two-dimensional cephalometric analysis and dental casts mounted on the articulator with a facebow transfer of the patient’s occlusal plane, and manual model surgery is performed to predict the direction and extent of movement in the jaw bone segment [1]. Several sources of error and inaccuracy are associated with the whole cranial situation in plaster model surgery because of insufficient control in rotation and the translation of movements [2,3]. To date, many methods have taken advantage of 3D digital models to improve overall procedures in orthognathic surgery and provide more efficient, affordable, predictable, and convenient planning and simulation [4,5,6,7,8,9,10,11,12]. Surgical planning and 3D printing methods based on 3D digital models have higher accuracy in osteotomy and repositioning and are less time-consuming in comparison with conventional methods [13,14]. Surgical outcomes after the use of a 3D printing splint show similar accuracies to those by wafers produced from conventional model surgery [14,15], and accurate, predictable, and efficient treatment outcomes can be achieved using 3D virtual planning [16].

In computer-assisted orthognathic surgery planning and simulation using digital 3D models, massive image data need to be transferred and shared between clinicians and other relevant professionals for collaboration. It is essential to share the same data conveniently during surgical planning and simulation for efficient collaboration between clinicians and technicians [17]; however, the use of local planning and simulation software installed on a desktop PC has some significant drawbacks during collaboration, for instance inefficiency in the transfer of image data, difficulty in sharing large data, and installation or maintenance of the software. This causes a lack of efficiency and increases the time and costs involved in data sharing and collaboration for surgical planning and simulation. To overcome these drawbacks, Schendel et al. developed a web-based simulation system for craniofacial surgery planning, which increased the availability and decreased costs [18]. The web-based system allowed the interaction of multiple users at a distance and the immediate dissemination of the application [18]. In addition, cloud-based applications can allow not only the convenient sharing of 3D digital models between clinicians and technicians, but can also allow easy collaboration in the planning process.

Fully digitalized orthognathic surgery planning and simulation requires the reproduction of the natural head positions (NHPs) of patients instead of the facebow transfer used in conventional model surgery [19]. The NHP, a standardized and reproducible position of the head in an upright posture, can be used as a reference coordinate in digital surgery planning using 3D models [20]. Instead of the intracranial reference, which is subject to considerable biological variation, the NHP provides the key for meaningful cephalometric analysis [21]. Previously, we developed a 3D digital NHP reproduction method; the NHP was applied to digital skeletal models for planning and simulation of orthognathic surgery in a local application [15,22]. To date, we are not aware of any studies that have applied the 3D digital model with NHP to a cloud-based application for planning and simulation in orthognathic surgery.

In this study, we developed a complete digital workflow for planning, simulation, and evaluation for orthognathic surgery based on 3D digital NHP reproduction, a cloud-based collaboration platform for planning and simulation, and 3D landmark-based evaluation, and evaluated the accuracy of orthognathic surgery performed using the digital workflow.

## 2. Materials and Methods

### 2.1. Acquisition of Patients’ CT and 3D Dentition Data

We included 24 patients (12 women and 12 men; mean age 22.08 ± 4.25 years) with skeletal class II and III malocclusion who underwent bimaxillary surgery at the Seoul National University Dental Hospital. We included patients with cloud-based digital surgical planning and simulation, use of 3D printing splint by digital surgical planning, and preoperative and immediate postoperative (1–6 weeks) CT data, and excluded patients with anomalies such as cleft lip or palate, hemifacial microsomia, and other syndromic diseases. The patient data were obtained with multi-detector CT (SOMATOM Sensation 10, Siemens, Munich, Germany) at 120 kVp and 80 mA, with a slice thickness of 0.75 mm. This study was performed with approval from the institutional review board of Seoul National University Dental Hospital (CRI12017).

To obtain skeletal models with artifact-free, high-resolution dentitions, the dental plaster casts of the patients were scanned using an optical model scanner (Maestro 3D, Maestro, Pisa, Italy) [23] and the scanned dentitions were fused with the corresponding maxillary and mandibular models using a modified iterative closest point (ICP) algorithm combined with a coherent point drift (CPD) algorithm [24,25,26,27,28]. A k-dimensional tree algorithm was used to accelerate and optimize the ICP algorithm process used for finding the closest points in the corresponding models by searching for the nearest neighbors [29]. After the initial matching using the ICP, we applied the CPD to refine the initial alignment. The fused digital skeletal models with artifact-free high-resolution dentitions were saved as stereolithography (standard triangulated language, STL) files in a cloud-based storage platform for further processing.

### 2.2. Cloud-Based Platform for Collaboration

The cloud-based application and data storage platform consisted of three sections: a client-side application, a server-side application, and private cloud data storage. On the client-side application, we applied HTML5, JavaScript, Bootstrap, and Three.js to display the patients’ 3D models on the web browser in the web interface development. Three.js was accessed via the HTML5 Canvas element using a document object model interface. JavaScript was used to accomplish the client-side browser in order to control and alter the displayed content. On the server-side application, we applied the Apache HTTP server to respond to web requests on the webserver. The 3D digital skeletal models of the patients were stored and shared via private cloud data storage using NAS (NAS4, EFM-Networks, Yongin, Korea), where only authorized users could access the data. Accordingly, the clinicians and other specialists could collaborate with the 3D massive skeletal model data based on the cloud-based platform using any computer or mobile device via the Internet anytime, anywhere.

### 2.3. Reproduction of the 3D NHP Model Based on Cloud-Based Storage

**Figure 1:**
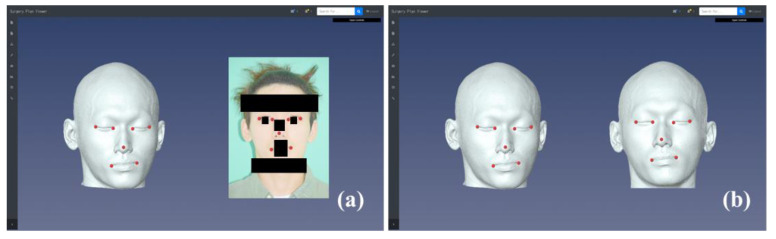
Reproduction of the 3D NHP model on the cloud-based platform (**a**) and comparison of the 3D CT models before and after natural head position (NHP) reproduction (**b**).

Previously, we developed an NHP reproduction method based on a pose from orthography and scaling with iterations (POSIT) algorithm using a single facial photograph [22]. In the study, the camera and CT coordinate systems coincided using the global coordinate system, as the CT scanner was typically positioned in the global coordinate system. The method determined the 3D rotation matrix using the feature points designated in the 2D photograph image and the corresponding 3D CT model based on the POSIT algorithm [22]. The landmarks used for NHP reproduction were the pronasale, bilateral exocanthion, endocanthion, and cheilion (Figure 1). The 3D rotational matrix was applied to the CT skeletal model to reproduce the CT model with NHP, then the NHP CT skeletal model was uploaded to a cloud-based storage platform as an STL file for sharing. The virtual osteotomy was performed with Geomagic Freeform software (Geomagic Inc., Morrisville, NC, USA) after importing the NHP CT model from storage. The virtual maxillary and mandibular distal and proximal segment models were generated by cutting the NHP CT model according to the planes used in actual surgery. After finishing the virtual osteotomy, the digital skeletal models were uploaded to the cloud-based storage platform as STL files for sharing.

### 2.4. Cloud-Based Collaboration for Surgical Planning and Simulation

**Figure 2:**
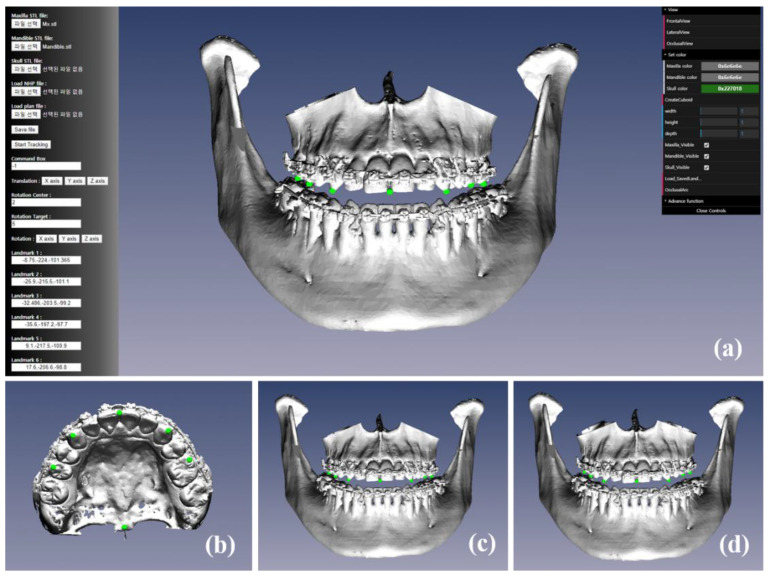
Cloud-based platform for orthognathic surgery planning and simulation (**a**), landmarks on the maxillary skeletal model with high-resolution and artifact-free dentitions (**b**), and the preoperative (**c**) and simulation (**d**) maxillomandibular complex (MMC) models and landmarks.

Previously, we developed methods for planning and simulation for orthognathic surgery using digital skeletal models with NHP in a local application [15,22]. In the present study, the skeletal models were downloaded from cloud-based data storage as STL files and were imported into the cloud-based application for planning and simulation on the web browser (Figure 2). The landmarks used for the displacement were designated on the skeletal model with high-resolution and artifact-free dentitions, then the desired amount of displacement was entered using a graphic user interface (Figure 2a,b). The bone segment was translated linearly along the left–right (*x*-axis), advance–setback (*y*-axis), and impaction–elongation (*z*-axis) directions using a given amount of displacement, then rotated around the rotation center by the angle of rotation in degrees determined between the target and goal points based on the method that we had developed previously [15,30]. After finishing the planning using the skeletal model, the total planned translational and rotational displacements were applied to the preoperative maxillomandibular complex (MMC) model and landmarks (Figure 2c), which simulated their final positions (Figure 2d). Both the simulation model and displaced landmarks were verified interactively by zooming, rotating, and translating the digital model.

**Figure 3:**
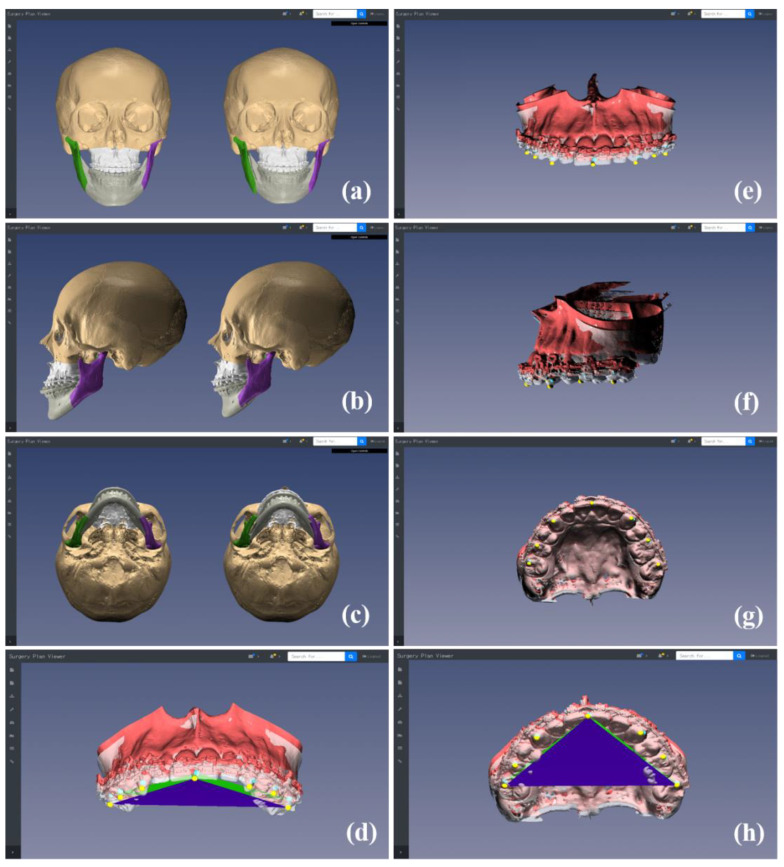
Frontal, lateral, bottom, and custom camera views used for reviewing simulation models and landmarks: comparisons between the preoperative and simulation maxillomandibular complex (MMC) models (**a**–**c**), between the preoperative (model in gray and landmarks in yellow) and simulation (model in red and landmarks in cyan) maxillary models and landmarks (**e**–**g**), and between the preoperative (in blue) and simulation (in green) occlusal plane inclinations (**d**,**h**) in different views.

**Figure 4:**
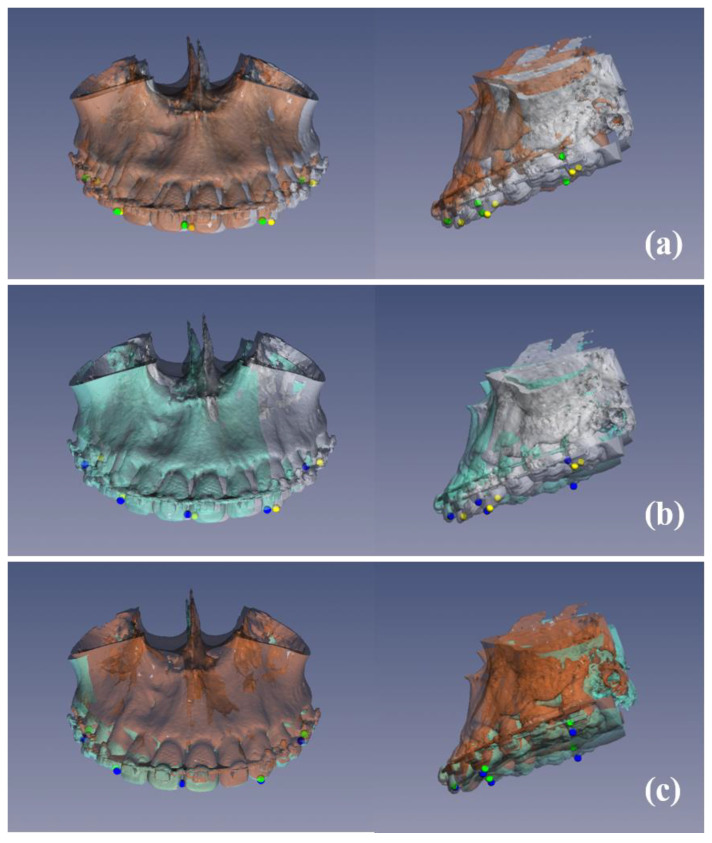
Multiple simulation models and landmarks generated from multiple surgical plans. Three representative cases of simulation models generated from multiple plans (**a**–**c**).

The clinician could review the simulation models and landmarks in frontal, lateral, bottom, and custom camera views, and could examine the changes between preoperative and simulated MMC models and between occlusal plane inclinations by controlling the viewing posture of the models on the web browser (Figure 3). The multiple simulation models could be generated from multiple surgical plans for a specific patient with severe facial asymmetry or deformity (Figure 4). The clinician could compare the different simulation models in detail via the superimposition of the simulation models and landmarks to ensure optimal planning (Figure 4). The optimal simulation model could be selected among models generated by multiple plans for better surgical results. After finishing the planning and simulation, the final MMC model was uploaded on the cloud-based storage platform as STL files.

Then, digital surgical splints were designed and generated by applying a Boolean operation to the dentitions from the simulated MMC model using Geomagic Freeform (Geomagic Inc.). Finally, the digital splint models were transferred to a 3D printing center via the Internet and physical splints were fabricated using a 3D printer (ProJet 7000, 3D Systems Inc., Rock Hill, SC, USA). Through the cloud-based platform, the surgeon and engineer could modify the digital surgical splint cooperatively and repeatedly for better surgical outcome before or after the fabrication of the physical splint. The engineer could redesign the digital splint model before fabrication if the splint design had to be modified according to the revised plan from a surgeon and after fabrication if the physical splint did not fit well with the patient’s dentition.

### 2.5. Comprehensive Evaluation Using Identical 3D Landmarks on Simulation and Postoperative Models

First, to evaluate the accuracy of the orthognathic surgery performed using the splints produced by the fully digitalized surgical planning and simulation model, we performed registration between simulation and postoperative CT models in the cranial bases of non-surgically exposed regions by surface-based registration using the same modified ICP algorithm [26,27]. Then, we calculated the translational and rotational discrepancies between 3D landmarks on the simulation and postoperative CT models.

The dentitions that had been previously fused with the preoperative skeletal model at the simulation stage were again fused with the postoperative CT model using the modified ICP at the evaluation stage; therefore, because the same dentition was registered with the preoperative and postoperative CT models, the identical 3D landmarks selected previously on the simulation model could be reused on the postoperative CT model without reselection of the landmarks.

The translational (linear) discrepancy between the simulation and postoperative CT models was quantified at eight landmarks in the bilateral incisors, canines, first molars, and second molars of the maxillary dentition. We calculated the absolute differences between landmarks in the left–right (*x*-axis), advance–setback (*y*-axis), and impaction–elongation directions (*z*-axis) and the root mean square (RMS) differences. To quantify the rotational (angular) discrepancy, a maxillary occlusal plane was defined using three landmarks on the mesiobuccal cusp of the first upper molar on the right side, on the left side, and the central incisors (Figure 3d,h) [31,32]. The rotational discrepancies between occlusal planes of the simulation and postoperative models were calculated in the pitch (around the *x*-axis), roll (around the *y*-axis), and yaw (around the *z*-axis) orientations.

The Kolmogorov–Smirnov test was performed to test the normality of the data distribution. Statistical analysis was performed using ANOVA and Pearson’s correlation coefficient using IBM SPSS software version 25 (IBM Corp., Armonk, NY, USA). We compared the absolute translational (linear) discrepancies between simulation and postoperative models in the left–right (*x*-axis), advance–setback (*y*-axis), and impaction–elongation directions (*z*-axis); the RMS translational (linear) discrepancies among eight landmarks; and the absolute rotational (angular) discrepancies between two models in pitch (*x*-axis), roll (*y*-axis), and yaw (*z*-axis) orientations using ANOVA tests. We also calculated Pearson’s correlation coefficients to determine the relationships between the displacements of surgical planning and discrepancies of surgical outcome.

## 3. Results

**Figure 5:**
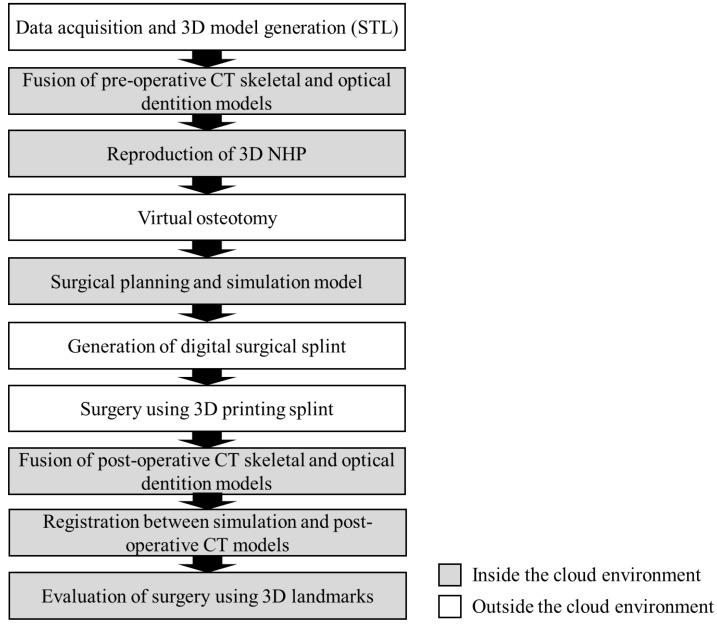
Overall procedure used for orthognathic surgery using a 3D printing splint with fully digitalized planning, simulation, and evaluation. When the process was performed outside the cloud environment, the STL data, which had no personally identifiable information, were transferred from NAS storage to the local workstation.

Figure 5 shows the overall procedure for orthognathic surgery using the 3D printing splint for fully digitalized planning, simulation, and evaluation.

**Figure 6:**
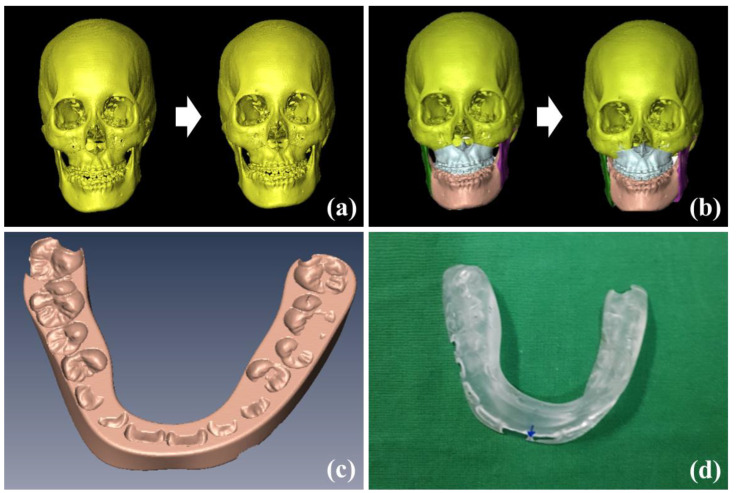
Digital models before and after natural head position (NHP) reproduction using the POSIT method (**a**), maxillomandibular complex (MMC) models before and after orthognathic surgery planning and simulation (**b**), digital surgical splint model generated from simulation maxillomandibular complex (MMC) models (**c**), and a physical splint fabricated via 3D printing (**d**).

Table 1 summarizes the surgical planning displacements for 24 patients who underwent bimaxillary surgery and details the left–right (*x*-axis), advance–setback (*y*-axis), and impaction–elongation (*z*-axis) translations, as well as the pitch (*x*-axis), roll (*y*-axis), and yaw (*z*-axis) orientations. Figure 6a shows the digital models of the patients before and after NHP reproduction using the POSIT method, while Figure 6b shows a simulation of the MMC models of the patients after planning. Figure 6c shows the digital surgical splints generated from the simulation MMC models, while Figure 6d shows the physical splints fabricated for the patients via 3D printing.

**Figure 7:**
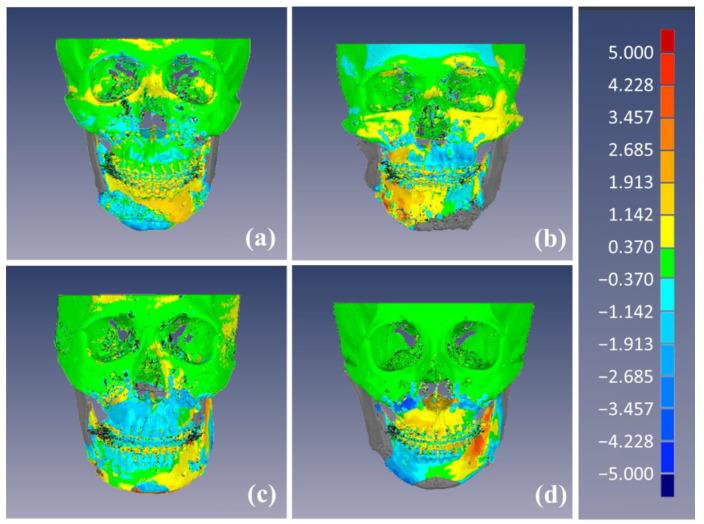
Discrepancies between the simulation and postoperative CT models as a color map (mm). Patients with the lowest linear discrepancy values in root mean square (RMS) (**a**), left–right (*x*-axis) (**b**), advance–setback (*y*-axis) (**c**), and impaction–elongation (*z*-axis) (**d**) directions.

After surgery, the postoperative CT models were matched to the simulation models via the registration process. Figure 7 shows the color mapping of the discrepancies between the simulation and postoperative CT models.

The absolute linear discrepancies at the eight tooth landmarks are summarized in Table 2. The means of the absolute linear discrepancies at the eight tooth landmarks were 0.61 ± 0.55, 0.86 ± 0.68, and 1.00 ± 0.79 mm in the left–right (*x*-axis), advance–setback (*y*-axis), and impaction–elongation (*z*-axis) directions, respectively, and 1.67 mm in the RMS direction. The absolute linear discrepancy in the left–right direction was significantly different from the other two directions (advance–setback and impaction–elongation) as shown by ANOVA (*p* < 0.05), while the RMS linear discrepancies showed no significant differences among the eight landmarks (ANOVA, *p* > 0.05) (Table 2).

**Figure 8:**
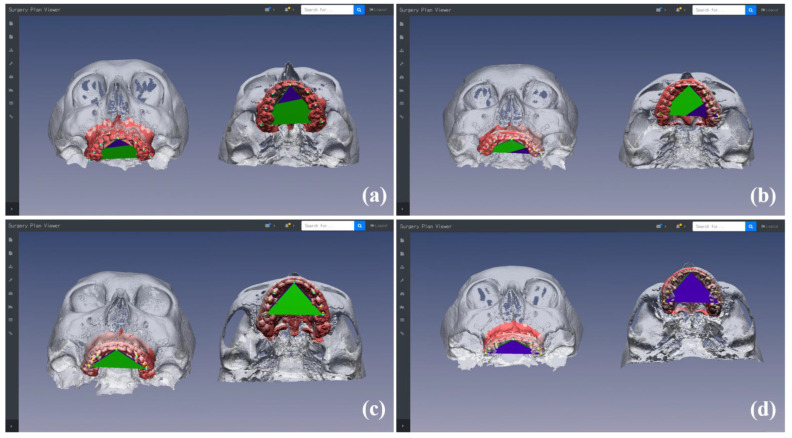
Rotational changes between simulation and postoperative CT models in occlusal planes and landmarks after surgery. Patients with the lowest linear discrepancy values in the root mean square (RMS) direction (**a**) and the lowest angular discrepancies in pitch (around *x*-axis) (**b**), roll (around *y*-axis) (**c**), and yaw (around *z*-axis) (**d**) orientations.

Figure 8 shows the rotational changes between simulation and postoperative CT models in occlusal planes and landmarks after surgery. The means of the absolute angular discrepancy were 1.43 ± 1.06°, 0.50 ± 0.31°, and 0.58 ± 0.41°, respectively, in the pitch (around the *x*-axis), roll (around the *y*-axis), and yaw (around the *z*-axis) orientations. The absolute angular discrepancy in the pitch orientation was significantly different from the other two orientations (roll and yaw) as shown by ANOVA (*p* < 0.05).

**Figure 9:**
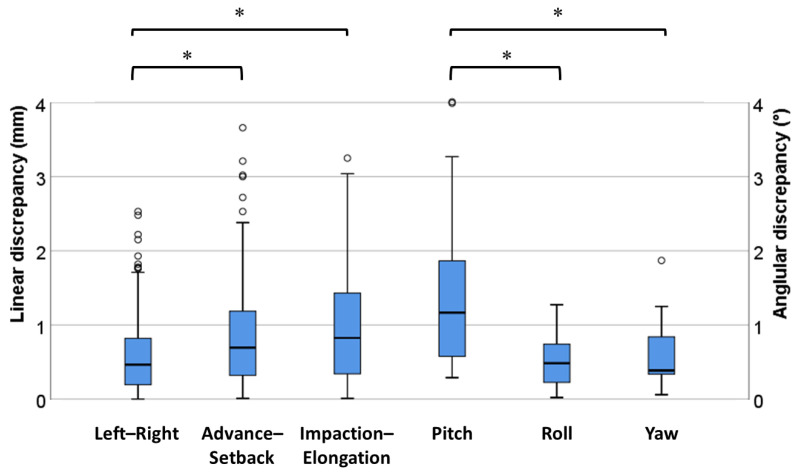
Boxplots of the linear and angular discrepancies between simulation and postoperative computed tomography models (*; *p* < 0.05).

The boxplot demonstrates the linear and angular discrepancies between simulation and postoperative models and describes the significant differences (Figure 9).

The advance–setback displacement of the surgical planning was positively correlated with the advance–setback translational discrepancy of the surgical outcome (*p* < 0.01) (Table 3). The yaw displacement of the planning was negatively correlated with the yaw rotational discrepancy of the outcome (*p* < 0.05) (Table 3).

## 4. Discussion

Compared with conventional orthognathic surgery, computer-assisted orthodontic and surgical analysis using 3D digital models has improved the effectiveness of the treatment planning process by eliminating previous procedures such as the mounting of casts on the articulator or the cutting and gluing of the casts [10]. Some studies have noted the advantages of computer-assisted techniques in predicting possible difficulties and complications [7] and enabling the precise visualization of osteotomized segments and the calculation of bony interferences [11]. Some researchers have reported the advantages of web- and cloud-based technologies for surgical planning and simulation in craniomaxillofacial surgery [18], orthognathic surgery [33], and other medical uses [17,34]. In one study, a cloud-based collaboration platform was proposed for orthognathic surgical planning; however, further studies are required to integrate more digital procedures, such as digital NHP reproduction and surgical outcome evaluation, for clinical validation [33]. As far as we know, this is the first study that uses a 3D digital NHP reproduction cloud-based collaboration platform for planning and simulation and a 3D landmark-based comprehensive evaluation for orthognathic surgery. Our study has provided a complete digital workflow for orthognathic surgery and has demonstrated its clinical efficacy.

We have developed a fully digitalized workflow for orthognathic surgery based on a cloud-based platform with efficient data sharing and close collaboration. First, we have implemented easy and convenient digital NHP reproduction with the POSIT algorithm using a single facial photograph, which showed clinically acceptable levels of accuracy of −0.04 ± 0.15°, −0.17 ± 0.50°, and −0.02 ± 0.37° in the roll, pitch, and yaw directions, respectively [22]. Then, the 3D digital skeletal models were shared between clinicians and co-workers via cloud-based data storage. The shared data as STL files, which had no personally identifiable information, could be accessed only by clinicians and technicians who were authorized by the institutional review board of Seoul National University Dental Hospital. The cloud-based storage platform was built in the hospital’s internal network and the data were not encrypted. Second, cloud-based surgical planning and simulations were performed on the web browser using a cloud-based application, which allowed immediate and convenient sharing of the massive simulation data without the installation or maintenance of the application. The optimal simulation model could be selected among models generated from multiple plans for better surgical results through close collaboration between clinicians and engineers. Surgeons sometimes require intraoperative modification of the surgical planning to consider facial symmetry, skeletal harmony, or the collision of ramus segments [9]. Then, the modified simulation model produced by an engineer using the revised plan from a surgeon can be shared immediately via the cloud-based platform. The surgeon can observe the modified simulation model on the screen in the operating room and can perform the surgery according to the modified plan without refabrication of the surgical splints. The complex simulations and operations might be difficult and inconvenient in terms of collaborative modification of the surgical planning using only software programs involving screen sharing or remote desktop connections. The surgeon and engineer could modify the digital surgical splint cooperatively and repeatedly before or after fabrication of the physical splint through the cloud-based platform. Third, we comprehensively evaluated the accuracy of the surgery performed by the 3D printing splint generated through the digitalized workflow using identical 3D landmarks on the simulation and postoperative CT models with automatic registration. The identical 3D landmarks selected on the dentition of the simulation model could be reused on the postoperative CT model without reselection of the landmarks on the postoperative model, which minimized human errors caused by the manual selection of landmarks during evaluation.

In this study, the mean RMS translational (linear) discrepancy between simulation and postoperative models was 1.67 mm at eight tooth landmarks for 24 patients, which was consistent with the clinically accepted difference of 2 mm for orthognathic surgery [35]. In one study, the error was within 1 mm and the maximum error was 1.7 mm at six landmarks on the maxilla for six patients using a CAD/CAM surgical guide consisting of osteotomy and repositioning guides [36]. Another study also reported less than 2 mm of the linear difference between reference points on the maxilla [37]. The linear discrepancies of 0.86 and 1.00 mm in the advance–setback (*y*-axis) and impaction–elongation (*z*-axis) directions, respectively, were larger than 0.61 mm in the left–right direction (*x*-axis) in our study. The smallest linear discrepancy was along the left–right direction (*x*-axis). In another study, the mean absolute difference of 1.1 mm in the impaction–elongation direction was larger than the 0.5 and 1.0 mm in left–right and advance–setback directions [37]. Similar results regarding the larger vertical error were also reported in previous studies [19,38]. Considering the aesthetic outcome of the upper lip, which was related to displacements of the midline (center of the incisors) in the left–right direction, the linear discrepancy in the impaction–elongation direction could be ignored when determining the appropriate position of the maxilla [38]. In addition, the means of absolute translational discrepancies showed a tendency to increase from the anterior tooth to the posterior tooth in the left–right and the anterior/posterior directions, but showed similar values in the impaction–elongation direction (Table 2). This caused some outliers in the left–right and anterior/posterior directions (Figure 9).

The angular discrepancy of 1.43° in the pitch orientation (around the *x*-axis) was larger than 0.50° in the roll (around the *y*-axis) and 0.58° in the yaw (around the *z*-axis) orientations, which might have been caused by the inevitable autorotation of the mandible that occurred when positioning the maxilla as a mandibular reference using a splint. Mandibular autorotation caused upward and forward movement of the jaw, resulting in advance–setback and impaction–elongation translations and pitch rotation. Rekow et al. reported the error in occlusion following splint removal and found an insignificant occlusal error, probably caused by the small amount of rotation taking place after splint removal [39,40]. Baan et al. also mentioned that these inaccuracies might have been associated with intraoperative changes in condylar position due to imperfect condylar seating [32]. Mandibular autorotation can cause differences of 10–20 mm between the condyle center and the mandibular rotation center, which can significantly affect the results of the orthognathic surgery, resulting in substantial errors in the position of the maxilla [39]. Additionally, the autorotation caused a relatively larger linear error in the advance–setback and impaction–elongation directions compared with the left–right direction.

Another reason for the larger discrepancies in the impaction–elongation direction and pitch orientation might be caused by the bony interferences between the pterygoid plates and the posterior part of the osteotomized maxilla [41]. This could lead to deviations in the impaction–elongation direction and pitch orientation, as it is difficult to visually check the bony interferences during surgery [41,42]. According to previous clinical studies on CAD/CAM splints, several factors could potentially have non-negligible impacts on the overall accuracy, which are (1) the final position of the maxilla, (2) image metal artifacts related to the plates, (3) errors related to the virtual mandibular autorotation, (4) the timing of the postoperative imaging, and (5) remodeling under muscular loading [43].

We also analyzed the relationships between the displacements of surgical planning and discrepancies of surgical outcome and found one positive and one negative relationship. The planned advance–setback displacement showed a positive correlation with the advance–setback outcome. As the advance–setback displacement was related to the shape of the lip and paranasal supporting function after surgery, it was possible to change the amount of displacement in the advance–setback direction for aesthetic and functional purposes during surgery, and the changed amount was proportional to the planned amount of the displacement [44]. On the other hand, the planned yaw displacement showed a negative correlation with the yaw outcome. The yaw displacement was related to the displacement in the left–right direction of the midline (center of the incisors), which was also closely related to the aesthetic outcome of the orthognathic surgery [38]. Since one of the most important objectives of orthognathic surgery is to improve the aesthetic outcome, the surgery should be performed to minimize the discrepancies between the planned displacements and the surgical outcomes in the yaw and left–right directions. A previous study reported that no significant correlations were found between the amount of planned advance–setback displacements and the mean differences and the mean absolute differences in six directions, including left–right, advance–setback, impaction–elongation, roll, pitch, and yaw directions [37].

In orthognathic surgery, close collaboration between the surgeon and other specialists is imperative through all stages of treatment, from preoperative planning to the finalization of occlusion [45]. The close interaction between orthodontists and surgeons is critical in devising and executing a comprehensive treatment plan successfully with predictable outcomes [45,46]. Surgeons also collaborate with engineers to simulate optimal surgical planning and fabricate 3D printing splints. In this interdisciplinary process, massive medical image data are shared among team members, such as orthodontists, surgeons, engineers, and 3D printing operators. The cloud-based collaboration platform for orthognathic surgery enabled immediate and convenient data sharing and close collaboration between the surgeon and other specialists, resulting in streamlined procedures through all stages of treatment. Although predicting the morphological changes of the facial soft tissue in response to orthognathic surgery was an important factor in improving the aesthetic outcome of surgery [47], the present cloud-based platform did not provide the cephalometric analysis based on the facial soft tissue landmarks or functions for predicting postoperative changes in the facial soft tissue. These functions would be particularly useful for evaluating perioperative changes in asymmetrical patients and developed based on artificial intelligence in future studies. Although our study was performed on patients undergoing conventional orthognathic surgery, we plan to recruit additional patients without presurgical orthodontic treatment and compare between outcomes using the conventional and the surgery first approaches [48].

To evaluate the accuracy of orthognathic surgery, we performed surface-based registration between the postoperative CT and simulation models in the cranial bases of a non-surgically exposed region using the modified ICP [26,27]. No statistical significance was found between surface- and voxel-based registrations in the study [49,50]; however, voxel-based registration was more accurate than surface-based registration, as the voxel-based registration relies on volumetric data rather than the surfaces after segmentation [32,51]. We comprehensively evaluated the translational and rotational accuracies at the identical 3D landmarks on the simulation and postoperative CT models by fusing the high-resolution and artifact-free dentition with preoperative and postoperative CT models, respectively, using automatic registration, which minimized the human error of manual landmark selection; however, the accuracy of the automatic registration method used in this study may influence the overall accuracy of the orthognathic surgery outcome. In addition, the patient’s dental model would be obtained directly from the patient’s dentition using a 3D intraoral scanner in order to avoid mistakes and imperfections when taking the impression and in the subsequent development of the plaster model.

## 5. Conclusions

In this study, the complete digital workflow based on the cloud-based platform provided streamlined procedures through all stages of orthognathic surgery, with efficient data sharing and close collaboration. The orthognathic surgery outcomes performed using the digital workflow showed high accuracy for the patients, which was comprehensively evaluated via the discrepancies between the 3D identical landmarks on the simulation and postoperative CT models. In future studies, we will automate and improve the essential tasks and functions required in the digital orthognathic surgery procedures using an artificial intelligent deep learning algorithm, such as localization of the 3D dental landmarks and segmentation of the maxilla, mandible, and teeth; however, the collaboration between the surgeon and other specialists will play a central role in better planning and management of the digital workflow in orthognathic surgery, which nowadays cannot be totally replaced by artificial intelligence.

## Figures and Tables

**Table 1:** Translational (mm) and rotational (°) displacements produced by the fully digitalized orthognathic surgery planning and simulation model for 24 patients.

Patients	Translation (mm)				Rotation (°)	
	Left –Right	Advance –Setback	Impaction –Elongation	Pitch	Roll	Yaw
1	3.44	0.19	2.98	0.00	−3.10	1.15
2	−1.38	1.05	0.59	2.82	3.35	−2.65
3	−1.72	0.12	−1.19	5.57	3.03	−3.33
4	0.69	−0.49	1.54	6.27	1.57	1.18
5	−0.24	1.12	−1.87	3.42	−4.67	0.00
6	−0.90	−2.76	5.41	8.31	3.22	0.00
7	0.38	1.88	3.41	8.22	0.00	−1.27
8	0.36	0.01	0.00	0.00	0.00	−1.14
9	2.20	1.90	1.63	1.83	3.02	0.00
10	0.72	−1.43	1.45	5.41	−0.13	−2.68
11	1.28	−0.28	−0.63	4.13	−4.68	2.41
12	−1.91	0.11	−1.09	7.37	1.56	0.00
13	−0.75	3.99	2.65	2.86	0.00	−1.10
14	1.63	−0.54	2.63	7.51	−1.44	2.84
15	−0.71	−0.14	−0.71	2.04	4.60	−1.65
16	2.09	1.14	2.86	4.20	1.51	0.00
17	0.57	0.13	−0.68	6.56	0.00	1.20
18	1.02	−0.01	1.30	4.47	−3.33	2.65
19	0.06	−0.12	−0.47	9.97	3.69	0.00
20	−1.11	1.14	−0.54	9.02	1.59	−2.69
21	0.77	0.09	−0.26	12.56	1.56	1.32
22	−1.32	2.32	−0.97	3.78	1.48	−2.23
23	−0.96	1.30	4.23	5.97	3.91	0.00
24	−1.50	−1.74	−1.05	9.28	0.00	−2.37
Mean ± SD	0.11 ± 1.40	0.37 ± 1.40	0.88 ± 1.98	5.48 ± 3.18	0.70 ± 2.63	−0.35 ± 1.79
Absolute Mean ± SD	1.16 ± 0.74	1.00 ± 1.01	1.67 ± 1.32	5.48 ± 3.12	2.14 ± 1.59	1.41 ± 1.10

**Table 2:** Means of absolute and root mean square (RMS) translational (linear) discrepancies (mm) between simulation and postoperative computed tomography models in left–right (*x*-axis), advance–setback (*y*-axis), and impaction–elongation directions (*z*-axis) at eight tooth landmarks for 24 patients after surgery (*; *p* < 0.05).

Landmarks	Left –Right	Advance –Setback	Impaction –Elongation	RMS
Right incisor	0.48 ± 0.49	0.79 ± 0.65	1.03 ± 0.84	1.58 ± 0.89
Left incisor	0.52 ± 0.49	0.83 ± 0.77	1.09 ± 0.91	1.67 ± 1.01
Right canine	0.45 ± 0.49	0.79 ± 0.56	1.05 ± 0.84	1.59 ± 0.81
Left canine	0.59 ± 0.38	0.96 ± 0.76	1.00 ± 0.90	1.72 ± 0.91
Right 1st molar	0.58 ± 0.44	0.83 ± 0.65	0.75 ± 0.52	1.42 ± 0.67
Left 1st molar	0.65 ± 0.56	0.88 ± 0.76	0.99 ± 0.79	1.72 ± 0.85
Right 2nd molar	0.90 ± 0.70	0.82 ± 0.43	1.07 ± 0.72	1.81 ± 0.72
Left 2nd molar	0.70 ± 0.62	1.00 ± 0.73	1.00 ± 0.67	1.82 ± 0.74
Mean ± SD	0.61 ± 0.55	0.86 ± 0.68 *	1.00 ± 0.79 *	1.67 ± 0.84

**Table 3:** Pearson’s correlation coefficients between displacements of surgical planning and discrepancies of surgical outcome (*; *p* < 0.5, **; *p* < 0.01).

Displacement by Planning	Translational Discrepancy			Rotational Discrepancy		
	Left –Right	Advance– Setback	Impaction– Elongation	Pitch	Roll	Yaw
Left–Right	0.09	−0.18	0.11	0.27	0.15	−0.12
Advance–Setback	−0.02	0.57 **	−0.32	0.20	0.04	0.00
Impaction–Elongation	0.30	−0.09	0.23	0.26	0.05	0.00
Pitch	−0.10	−0.02	0.13	−0.34	−0.16	−0.05
Roll	0.38	0.14	−0.23	0.04	−0.22	0.18
Yaw	−0.28	−0.01	0.31	−0.19	0.09	−0.41 *

## Data Availability

All data generated during the current study are not publicly available due to the restriction by the institutional review board (IRB) of the Seoul National University Dental Hospital in order to protect the patients’ privacy.
